# Cytokines and TLR agonists influence the expression of retention integrins CD49a, CD49b and CD103 by T cells

**DOI:** 10.1186/2051-1426-3-S2-P235

**Published:** 2015-11-04

**Authors:** Marit M Melssen, Ciara Hutchison, Ileana S Mauldin, Timothy N Bullock, Craig L Slingluff

**Affiliations:** 1 University of Virginia, Charlottesville, VA, USA

## 

T cell infiltration in the tumor microenvironment (TME) is positively correlated with survival. Infiltration is not a stable state, but a dynamic process of homing, infiltration, retention and survival. Every step in this process is crucial for the eventual tumor reactive activity of T cells. Our laboratory has previously shown a marked upregulation of retention integrins (RI) CD49a, CD49b and CD103 on tumor infiltrating lymphocytes (TIL) in melanoma. T cells in peripheral tissues expressing RI appear to have a cytotoxic phenotype and RI^+^ TIL are associated with improved patient survival. Despite their importance in retention of tumor reactive TILs, little is known about pathways inducing RI expression, except that TGFβ induces CD103. We hypothesized that other cytokines, TLR agonists and T cell receptor (TCR) stimulation can induce RI expression by circulating T cells. PBMCs were grown in presence of cytokines after TCR stimulation and RI expression was evaluated by flow cytometry after 2-7 days. Both CD103 and CD49a are induced by TGFβ, most strikingly on activated T cells. IL2 and IL10 also influence CD49a expression. CD49b expression is increased by IL2, IL4 and IL10, although this depends on T cell subset and activation state. TLR2, TLR6 and TLR7 agonists appear to induce CD49a and CD49b as well. In addition we have found that IL2 and TGFβ upregulate RI expression on T cells with different proliferation rates and cytotoxic activity. In conclusion, above results support our hypothesis by showing that RI expression is tightly regulated by cytokine and TCR stimulation. Interestingly, the regulating factors have different effects among the RI and phenotype of RI+ T cells. Which suggests a different role for each of the RI and a substantial contribution of the induction route in the determination of this role. Furthermore the cytokines found to be involved with RI expression are associated with either Th1, Th2 or Treg cells. All raising interesting questions about the features of the TME that best support tumor reactive T cell retention in the tumor.

